# Supplementary material for: The Glucose Transporter GLUT3 Controls Regulatory T Cell Function
Source: Eur J Immunol. 2026 Aug 24;56(8):e70272. doi: 10.1002/eji.70272 (PMC13501943; doi:10.1002/eji.70272)
Supplement: Supplementary file 1 [file EJI-56-e70272-s001.pdf]

SUPPLEMENTARY INFORMATION

The Glucose Transporter GLUT3 Controls Regulatory T Cell Function

Katrin Sinning, Miriam Eckstein, Xiufeng Zhao, Alexandra Freitag,
Mathias Rosenfeldt, Sophia M. Hochrein and Martin Vaeth

Supplementary Figures S1-S6

Supplementary Tables S1 and S2

Supplementary Figure S1

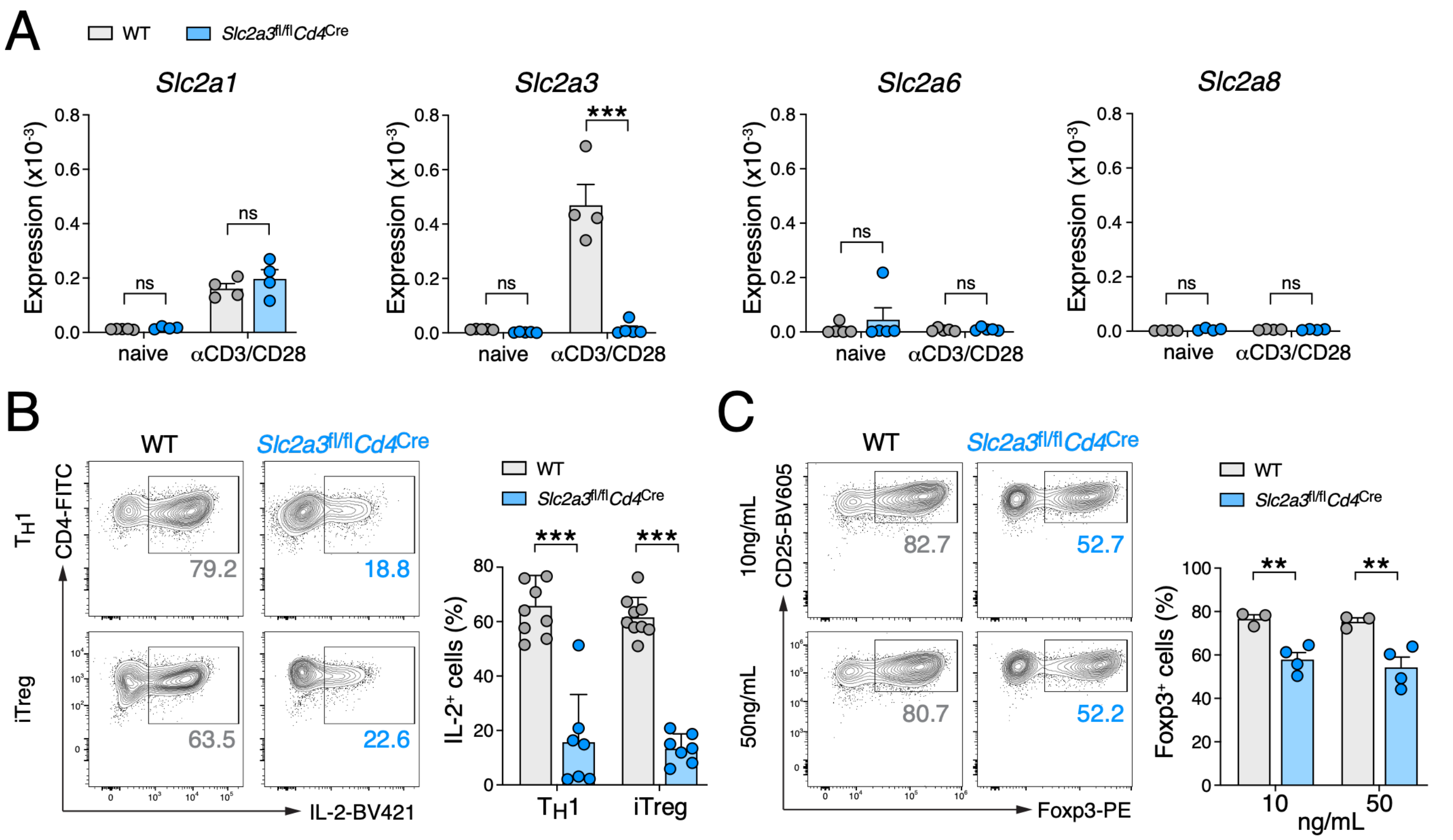

Figure S1. Ablation of GLUT3 in T cells impairs IL-2 production *in vitro*. (A) Analysis of *Slc2a1* (GLUT1), *Slc2a3* (GLUT3), *Slc2a6* (GLUT6) and *Slc2a8* (GLUT8) gene expression in naïve and anti-CD3/CD28-stimulated $CD4^+$ T cells using RT-qPCR; data shown as means ± SEM of 4 mice. **(B)** Analysis of intracellular IL-2 production in *in vitro*-differentiated Th1 and iTreg cells after re-stimulation with PMA/ionomycin by flow cytometry; means ± SEM 7-8 mice. **(C)** Differentiation of iTreg cells from naïve WT and GLUT3-deficient T cells with standard (10 ng/mL) and high dose (50 ng/mL) of recombinant IL-2; means ± SEM 3-4 mice. Statistical analysis in panels (A-C) was performed using two-way ANOVA. ***, p<0.001; ns, non-significant.

Supplementary Figure S2

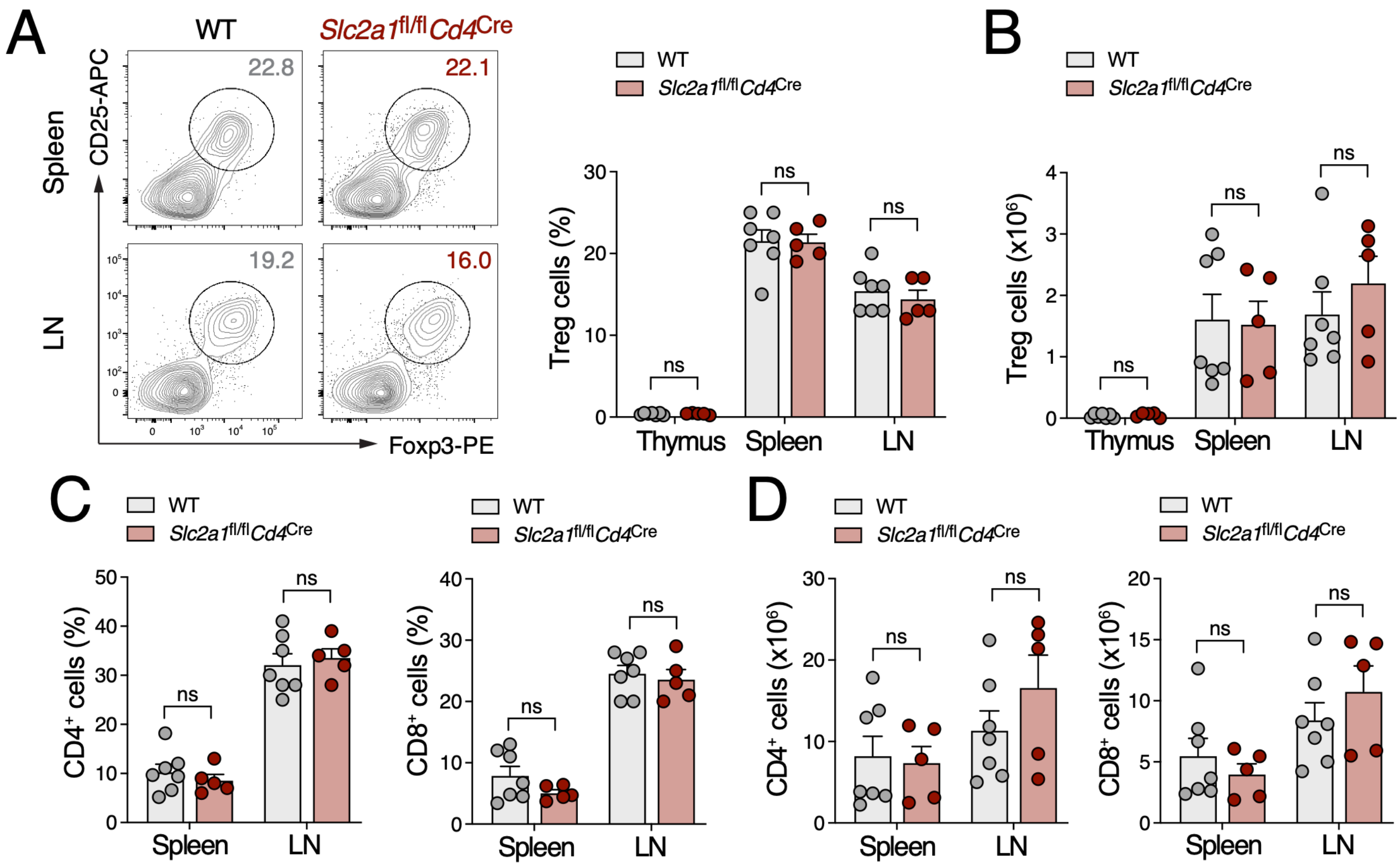

Deletion Ablation of GLUT1 in T cells does not alter Treg cell homeostasis in lymphoid organs. (A) Flow cytometric analysis of Foxp3$^+$ Treg cells in the thymus, spleen and lymph nodes (LNs) of WT and *Slc2a1*$^{fl/fl}$*Cd4*^{Cre} mice; data are shown as means ± SEM of 5-7 mice. **(B)** Quantification of absolute Treg cell numbers in the indicated lymphoid organs from WT and *Slc2a1*$^{fl/fl}$*Cd4*^{Cre} mice; means ± SEM of 5-7 mice. **(C and D)** Analysis of conventional CD4$^+$ and CD8$^+$ T cells in the spleen and LNs of WT and *Slc2a1*$^{fl/fl}$*Cd4*^{Cre} mice. Frequencies **(C)** and absolute cell numbers **(D)** are shown as means ± SEM from 5-7 mice per group. Statistical analysis in panels (A-D) was performed using two-way ANOVA. ns, non-significant.

Supplementary Figure S3

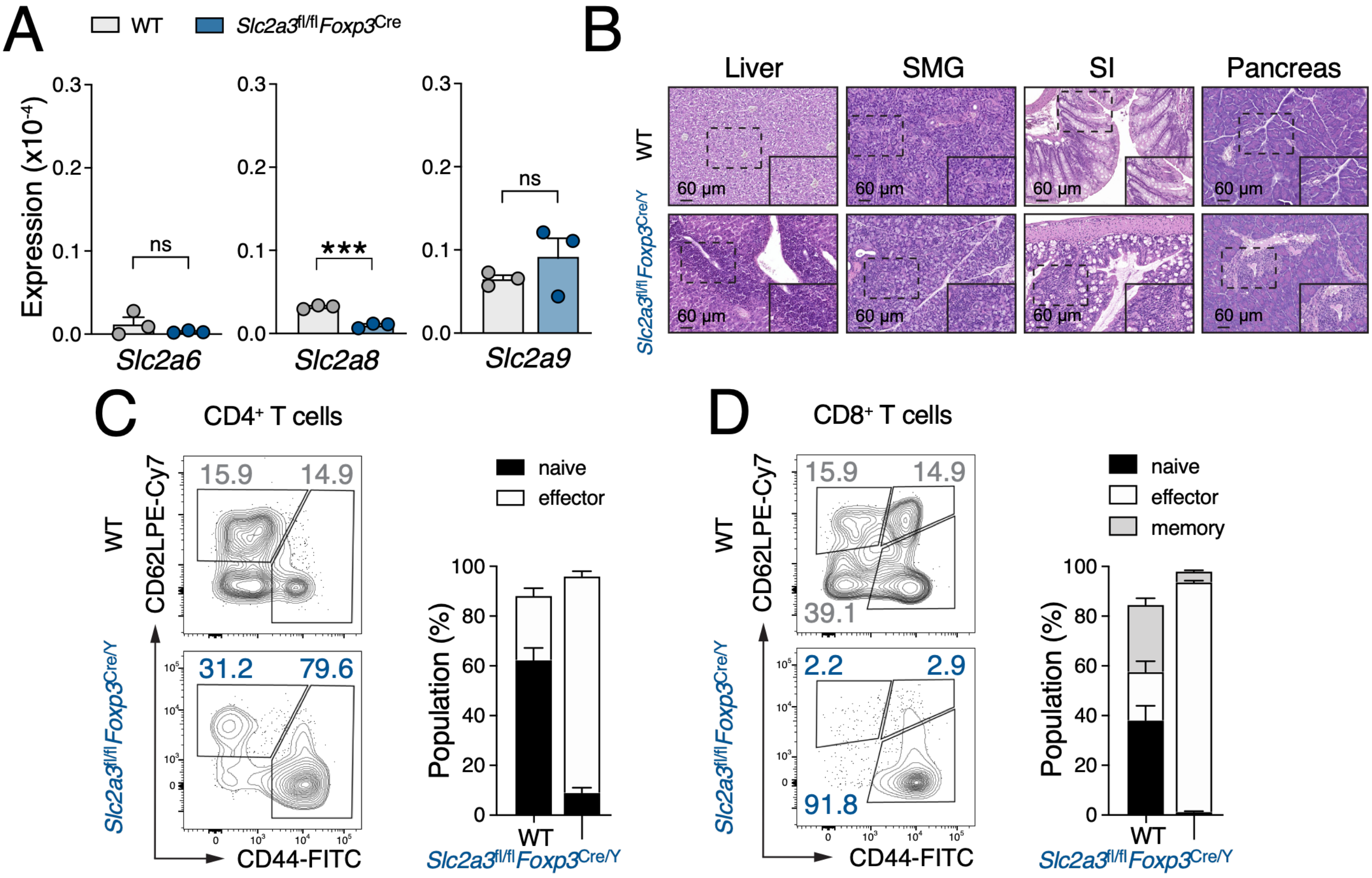

Figure S3. Treg cell-specific deletion of GLUT3 causes systemic immune activation and inflammation. (A) Analysis of *Slc2a6* (GLUT6), *Slc2a8* (GLUT8) and *Slc2a9* (GLUT9) gene expression in FACS-sorted Treg cells from WT and *Slc2a3*$^{fl/fl}$*Foxp3*^{Cre} mice by RT-qPCR; data are shown as means ± SEM of 3 mice. **(B)** Representative examples of hematoxylin- and eosin-stained sections of liver, submandibular glands (SMG), small intestine (SI) and pancreas from male WT and *Slc2a3*$^{fl/fl}$*Foxp3*$^{Cre/Y}$ mice; scale bars represent 60 µm. **(C and D)** Flow cytometric analysis of naïve (CD62L^{hi}CD44^{lo}), effector (CD62L^{lo}CD44^{hi}) and central memory (CD62L^{hi}CD44^{hi}) CD4^{+} **(C)** and CD8^{+} **(D)** T cells in the spleen of male WT and *Slc2a3*$^{fl/fl}$*Foxp3*$^{Cre/Y}$ mice; means ± SEM of 10-13 mice. Statistical analysis in panels (A) was performed using two-way ANOVA. ***, p<0.001; ns, non-significant.

Supplementary Figure S4

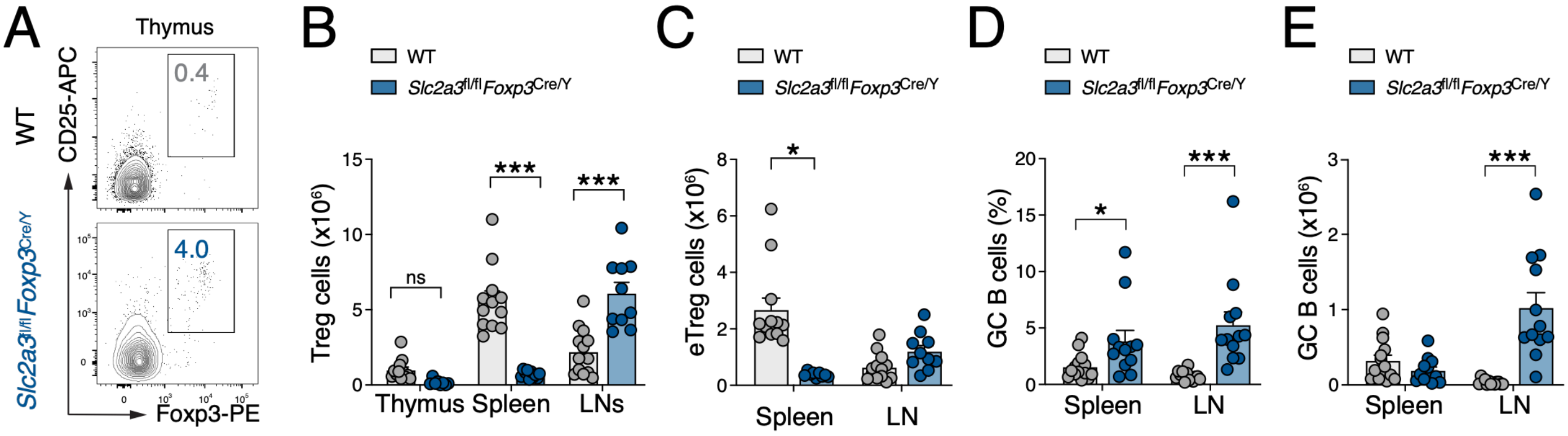

Figure S4. GLUT3 regulates effector Treg cell differentiation and function. (A and **B)** Quantification of relative **(A)** and total **(B)** Foxp3^{+} Treg cell numbers in the thymus, spleen and lymph nodes (LNs); data are shown as means ± SEM of 10-13 mice. **(C)** Quantification of effector Treg (eTreg) cell numbers in the spleen and LNs of male WT and *Slc2a3*$^{fl/fl}$*Foxp3*$^{Cre/Y}$ mice; means ± SEM of 10-13 mice. **(D** and **E)** Quantification of the frequencies **(D)** and total cell numbers **(E)** of germinal center (GC) B cells in the spleen and LNs of male WT and *Slc2a3*$^{fl/fl}$*Foxp3*$^{Cre/Y}$ mice; data are means ± SEM of 10-13 mice. Statistical analysis in panels (A-E) was performed using two-way ANOVA. *, p<0.05; **, p<0.01, ***, p<0.001.

Supplementary Figure S5

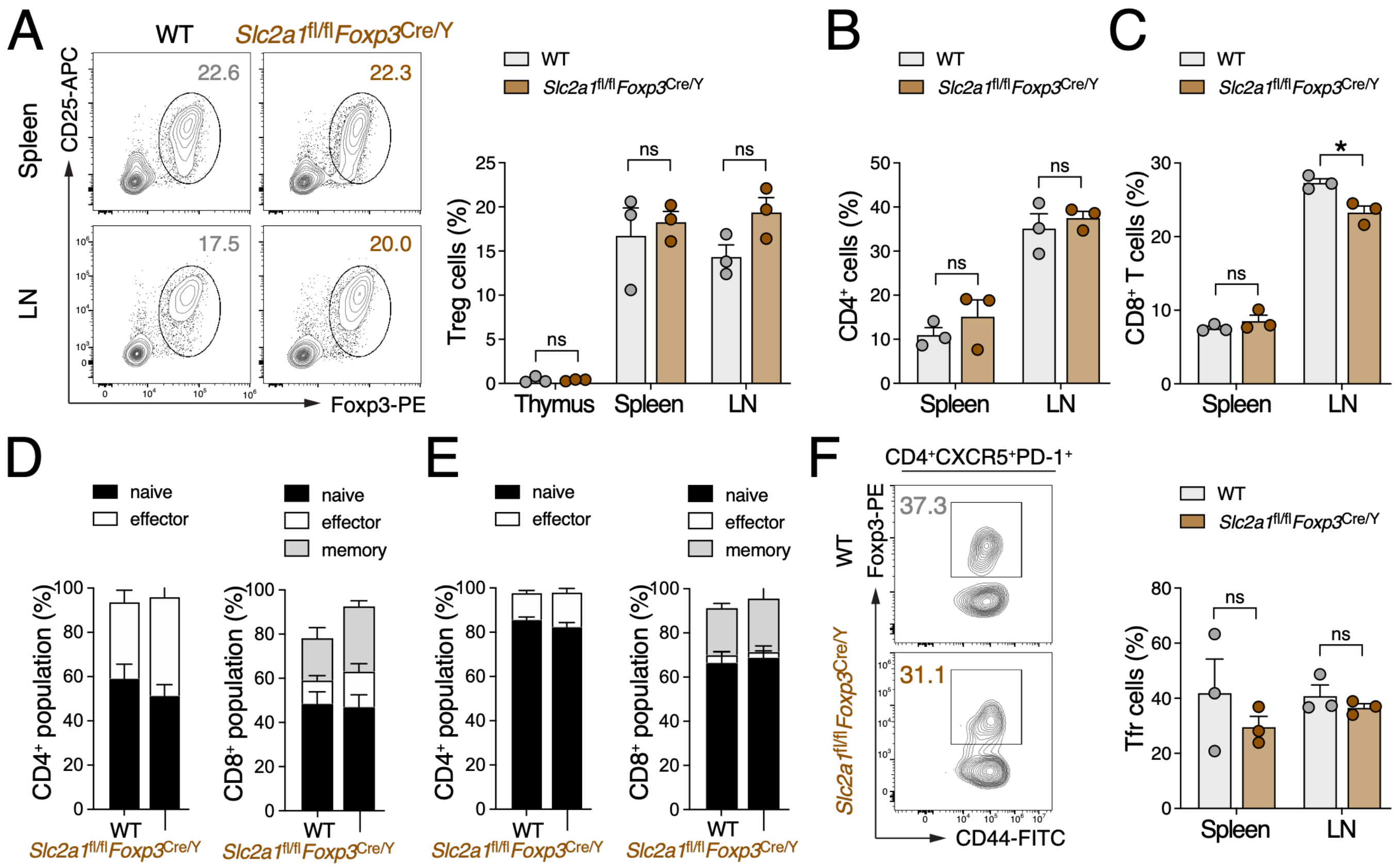

Figure S5. Treg cell-specific ablation of GLUT1 does not alter Treg homeostasis, immune composition and effector differentiation. (A) Flow cytometric analysis of Foxp3^{+} Treg cells in the spleen and lymph nodes (LNs) of hemizygous male WT and *Slc2a1*$^{fl/fl}$*Foxp3*$^{Cre/Y}$ mice; data are shown as means ± SEM of 3 mice. **(B** and **C)** Analysis of conventional CD4^{+} **(B)** and CD8^{+} T cells frequencies **(C)** in the spleen and LNs from WT and *Slc2a1*$^{fl/fl}$*Foxp3*$^{Cre/Y}$ mice; data are shown as means ± SEM of 3 mice. **(D** and **E)** Flow cytometric analysis of naïve (CD62L^{hi}CD44^{lo}), effector (CD62L^{lo}CD44^{hi}) and central memory (CD62L^{hi}CD44^{hi}) CD4^{+} and CD8^{+} T cells in the spleen **(D)** and LNs **(E)** of male WT and *Slc2a1*$^{fl/fl}$*Foxp3*$^{Cre/Y}$ mice; means ± SEM of 3 mice. **(F)** Representative flow cytometric analysis of T follicular regulatory (Tfr) cells in the spleen and LNs of male WT and *Slc2a1*$^{fl/fl}$*Foxp3*$^{Cre/Y}$ mice, with quantification of Tfr cell frequencies; means ± SEM of 3 mice. Statistical analysis in panels (A-E) was performed using two-way ANOVA. *, $p<0.05$; ns, non-significant.

Supplementary Figure S6

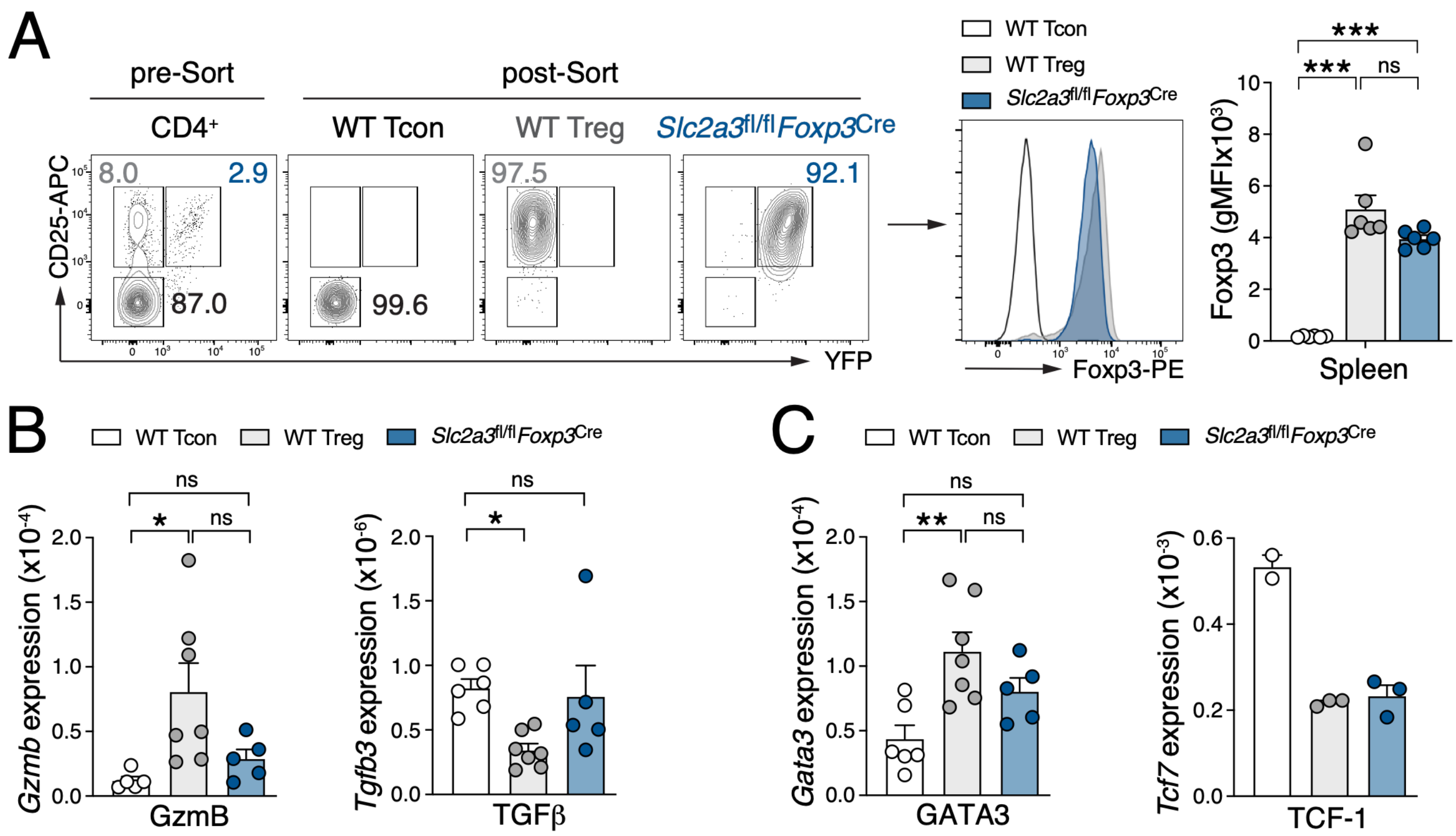

Figure S6. Isolation and characterization of WT and GLUT3-deficient Treg cells. (A) Representative FACS-sorting strategy to isolate T conventional (Tcon), WT and GLUT3-deficient Treg cells from female heterozygous *Slc2a3*$^{fl/fl}$*Foxp3*$^{Cre/+}$ mice (left) and verification of intracellular Foxp3 expression post sorting. **(B)** Expression of *Gzmb* (granzyme B) and *Tgfb3* (TGFβ) in FACS-sorted Tcon, WT and GLUT3-deficient Treg cells from female heterozygous *Slc2a3*$^{fl/fl}$*Foxp3*$^{Cre/+}$ mice measured by RT-qPCR; data shown as means ± SEM of 5-7 mice. **(C)** Expression of *Gata3* (GATA-3) and *Tcf7* (TCF-1) in FACS-sorted Tcon, WT and GLUT3-deficient Treg cells from female heterozygous *Slc2a3*$^{fl/fl}$*Foxp3*$^{Cre/+}$ mice measured by RT-qPCR; data shown as means ± SEM of 2-7 mice. Statistical analysis in panels (A) was performed using two-way ANOVA. *, p<0.05; **, p<0.01; ***, p<0.001; ns, non-significant.

Table S1. List of antibodies used in this study

Antibody	Clone	Company	Cat#	RRID	Dilution
anti-B220 (FITC conjugated)	RA-6B2	BioLegend	103206	AB_312991	1:400
anti-CD3 (PE conjugated)	145-2C11	BioLegend	100308	AB_312673	1:400
anti-CD3 (FITC conjugated)	145-2C11	BioLegend	100306	AB_312671	1:400
anti-CD4 (BV421 conjugated)	RM4-5	BioLegend	100544	AB_2563052	1:400
anti-CD4 (BV605 conjugated)	GK1.5	BioLegend	100451	AB_2564591	1:400
anti-CD8 (BV711 conjugated)	53-6.7	BioLegend	100748	AB_2563510	1:400
anti-CD11b (PE conjugated)	M1/70	BioLegend	101208	AB_312791	1:400
anti-CD19 (BV421 conjugated)	6D5	BioLegend	115538	AB_11203527	1:500
anti-CD25 (APC conjugated)	PC62	BioLegend	102012	AB_312861	1:400
anti-CD38 (APC conjugated)	90	BioLegend	102712	AB_312932	1:400
anti-CD44 (FITC conjugated)	IM7	BioLegend	103043	AB_2561391	1:400
anti-CD45 (BV510 conjugated)	104	BioLegend	109838	AB_2561393	1:400
anti-CD62L (PE-Cy7 conjugated)	MEL-14	BioLegend	104418	AB_313102	1:400
anti-CXCR5 (PerCP-Cy5.5)	SPRCL5	eBioscience	46-7185-82	AB_2561972	1:100
anti-Foxp3 (PE conjugated)	FJK-16s	ThermoFisher Scientific	12-5773-82	AB_465936	1:200
anti-Foxp3 (FITC/AF488 conjugated)	FJK-16s	ThermoFisher Scientific	53-5773-82	AB_763537	1:200
anti-Foxp3 (APC conjugated)	FJK-16s	ThermoFisher Scientific	17-5773-82	AB_469457	1:200
anti-F4/80 (BV421 conjugated)	BM8	BioLegend	123137	AB_2563102	1:400
anti-GFP (AF488 conjugated)	n/a	ThermoFisher Scientific	A-21311	AB_221477	1:100
anti-GM-CSF (PE conjugated)	MP1-22E9	BioLegend	505406	AB_315382	1:200
anti-GL7 (FITC/AF488 conjugated)	GL7	BioLegend	144612	AB_2563285	1:200
anti-IL-2 (BV421 conjugated)	JES6-5H4	BioLegend	503826	AB_2650897	1:200
anti-NK1.1 (FITC/AF488 conjugated)	PK136	BioLegend	108717	AB_493183	1:200
anti-PD1 (APC conjugated)	RMP1-39	BioLegend	109112	AB_10613470	1:400
anti-Siglec F (APC/AF647 conjugated)	E50-2440	BD Bioscience	562680	AB_2687570	1:400

Table S2. List of RT-qPCR primers used in this study.

Target	Forward	Reverse
18S	CGGCGACGACCCATTCGAAC	GAATCGAACCCTGATTCCCCGT
Slc2a1	GAGACCAAAGCGTGGTGAGT	GCAGTTCGGCTATAACACTGG
Slc2a3	ATCGTGGCATAGATCGGTTC	TCTCAGCAGCTCTCTGGGAT
Slc2a6	GCCCAGGAGGTCATTGAGTA	CCAGCACTACACCTGGACAA
Slc2a8	GGCATGTAGCACATGAGCAG	GGCATCCTCCTGGCCTAT
Slc2a9	TTGCTTTAGCTTCCCTGATGTG	GAGAGGTTGTACCCGTAGAGG
Gzmb	CCACTCTCGACCCTACATGG	GGCCCCCAAAGTGACATTTATT
Tgfb3	TCTCCACTGAGGACACATTGA	ATTCGACATGATCCAGGGAC
Gata3	AGGATGTCCCTGCTCTCCTT	GCCTGCGGACTCTACCATAA
Tcf7	AGCTTTCTCCACTCTACGAACA	AATCCAGAGAGATCGGGGGTC
Il10	TGTCAAATTCATTCATGGCCT	ATCGATTTCTCCCCTGTGAA
Ebi3	GGGATGCCAGAGCACCTG	TCCTAGCCTTTGTGGCTGAG
Ctla4	TTTTGTAGCCCTGCTCACTCT	CTGAAGGTTGGGTCACCTGTA
Hif1a	AAACTTCAGACTCTTTGCTTCG	CGGCGAGAACGAGAAGAA
Irf4	CAAAGCACAGAGTCACCTGG	TGCAAGCTCTTTGACACACA
Prdm1	TTCTCTTGGAAAAACGTGTGGG	GGAGCCGGAGCTAGACTTG
